# AMPSeek: A Workflow for Predicting Antimicrobial Peptide Activity, Three‐Dimensional Structure, and Toxicity

**DOI:** 10.1002/cpz1.70325

**Published:** 2026-02-19

**Authors:** Emily Zhang, Berke Ucar, Ali Salehi, René L. Warren, Inanc Birol

**Affiliations:** ^1^ BC Cancer Vancouver BC Canada; ^2^ Department of Medical Genetics University of British Columbia Columbia BC Canada

**Keywords:** antimicrobial activity, antimicrobial peptides, drug toxicity, machine learning

## Abstract

With antimicrobial resistance emerging as a top global health concern, new approaches to prevent and treat infections are urgently needed. Antimicrobial peptides are recognized as a promising alternative to conventional antibiotics. Considering the costly, labor‐intensive, and time‐consuming nature of wet lab screening, in recent years, proteomics research has increasingly relied on *in silico* methods to predict physicochemical, structural, and biological properties of candidate peptides. Here, we present the AMPSeek protocol to enable scientists to screen peptides and predict their potential antimicrobial activity, toxicity, and associated three‐dimensional structure with a single command. The AMPSeek framework integrates AMPlify, tAMPer, and LocalColabFold, scales efficiently with increasing sample size, and produces an interactive HyperText Markup Language report to facilitate result interpretation. We provide step‐by‐step instructions for installing and running AMPSeek on a ten‐peptide test set, along with two additional datasets (*n* = 20 and *n* = 100; mean peptide length ≈121.3) to demonstrate AMPSeek's near‐linear scalability. AMPSeek is available on GitHub under the GPLv3 software license agreement: https://github.com/bcgsc/AMPSeek. © 2026 The Author(s). Current Protocols published by Wiley Periodicals LLC.

**Basic Protocol**: Prediction of antimicrobial peptide activity, three‐dimensional structure, and toxicity

## INTRODUCTION

Antimicrobial resistance (AMR) is a growing global threat to public health (World Health Organization, 2023), accounting for 4.71 million documented deaths worldwide in 2021 and 169 million projected deaths by 2050 (Murray et al., 2022). Beyond human health, AMR also affects global economics, with an estimated decrease of 1.1% in global gross domestic product by 2050 when following current trends (World Bank, 2017). The decreasing effectiveness of traditional small‐molecule antibiotics due to AMR highlights a pressing need for the development of novel drugs. Antimicrobial peptides (AMPs), components of the innate immune system across multicellular organisms (Diamond et al., 2009), offer a promising alternative to traditional small‐molecule antibiotics. These short (typically 5‐50 amino acids long), amphipathic peptides exhibit broad‐spectrum antimicrobial activity against bacteria, fungi, and viruses through diverse mechanisms (Reddy et al., 2004; Zhang & Gallo, 2016), including membrane disruption and immune modulation (Hancock & Sahl, 2006). Furthermore, AMPs typically act faster than traditional antibiotics (Fantner et al., 2010), possess a narrower active concentration range for killing (Yu et al., 2018), avoid damaging the DNA of their targets, and have been shown to generate resistance in pathogens more slowly than conventional antimicrobials (Park et al., 2011; Rodríguez‐Rojas et al., 2014).

Historically, AMP discovery and evaluation involved costly, labor‐intensive, and time‐consuming wet lab screening methods (Wishart, 2019), hindering rapid progress in the field. In response, several *in silico* methods and tools have been developed to predict peptide activity (e.g., AMPlify; Li et al., 2022), structure (e.g., LocalColabFold; Mirdita et al., 2022), and toxicity (e.g., tAMPer; Ebrahimikondori et al., 2024), streamlining novel AMP discovery. These predictive algorithms, such as bidirectional long short‐term memory (bi‐LSTMs; Schuster & Paliwal, 1997), bidirectional gated recurrent units (bi‐GRUs; Cho et al., 2014), and graph neural networks (GNNs; Scarselli et al., 2009), are accurate, resource efficient, and fast relative to wet lab screening, and they can be used help narrow down a list of candidate putative AMPs before *in vitro* and *in vivo* validation.

**Figure 1:**
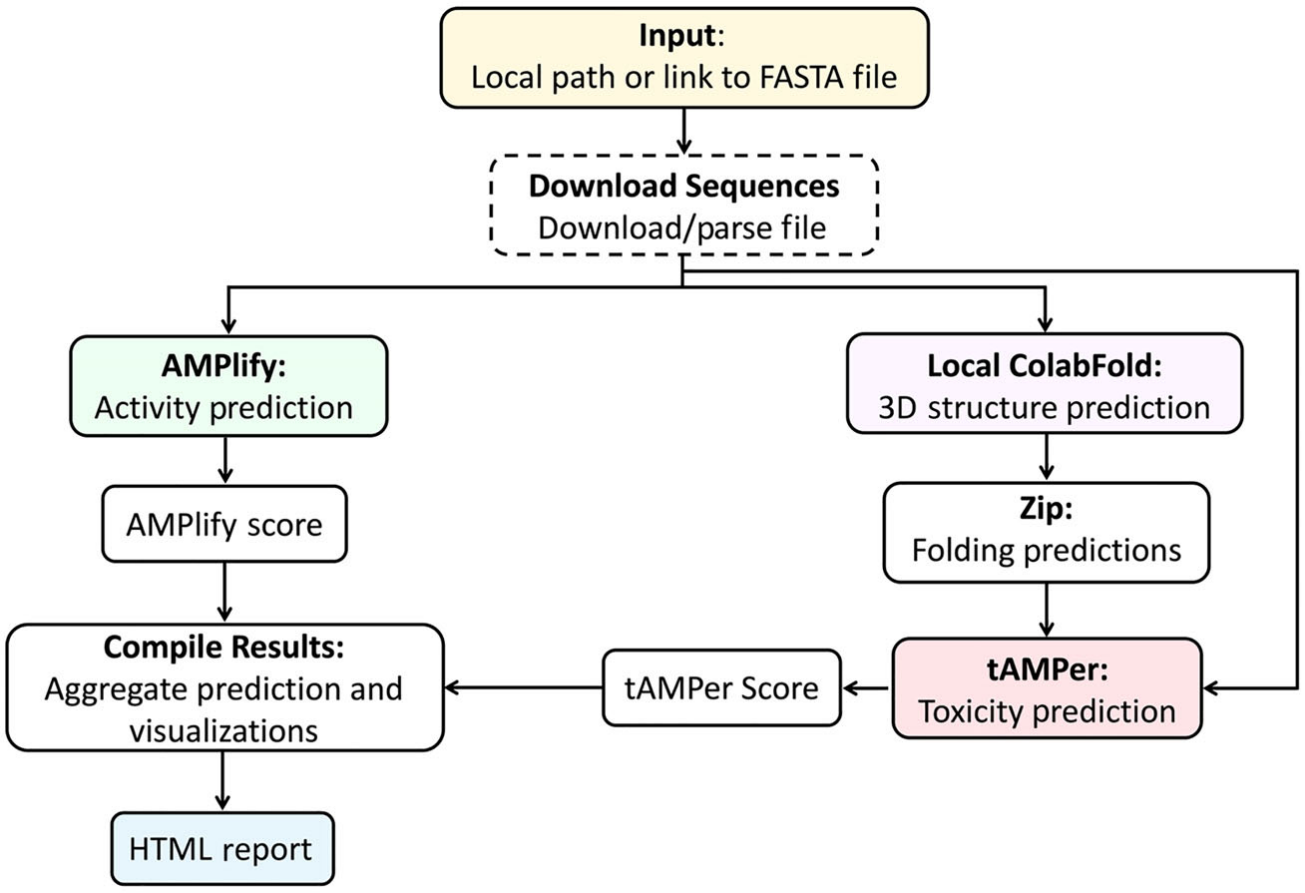
AMPSeek pipeline overview. Download Sequences downloads files from the internet if the ‐‐download_from flag is specified and parses them; this is an optional step, as denoted by the dashed outline. AMPlify produces an antimicrobial bioactivity score, Local ColabFold is used for three‐dimensional protein structure prediction of the putative AMP, tAMPer produces a hemolytic and cytotoxic activity score, and Compile Results aggregates the predictions into one HTML file with summary data visualizations

The protocol presented here, AMPSeek, first predicts bioactive peptides against a broad spectrum of pathogens with AMPlify, an attentive deep learning model that uses two types of attention mechanisms layered on top of a bi‐LSTM layer (Li et al., 2022). In parallel, LocalColabFold performs an MMseqs2 (many‐against‐many sequence searching)‐based homology search to accelerate AlphaFold2 (Yang et al., 2023) and predict the three‐dimensional structure of peptides (Mirdita et al., 2022). The unprocessed sequences and predicted structures are then analyzed with tAMPer to predict hemolytic and cytotoxic activity (toxicity) using bi‐GRUs, GNNs, and a self‐attention layer (Ebrahimikondori et al., 2024). Lastly, a graphical HyperText Markup Language (HTML) report is generated to summarize the results. These steps are performed using a single command at the Unix terminal, streamlining the AMP discovery process. A graphical overview of the workflow is shown in Figure 1.

We highlight the usage of the AMPSeek protocol using 10 peptides obtained from the AMPlify training set. We provide instructions for installing the required software in a container image in the Strategic Planning section. AMPSeek outputs a bioactivity score, a three‐dimensional structure prediction, and a toxicity score for each peptide, captured in an HTML report with simple data visualizations.

## STRATEGIC PLANNING

### Necessary Hardware

AMPSeek is a single‐protocol proteomics pipeline that can be run on 64‐bit Linux and Mac machines with sufficient available random‐access memory (RAM)—e.g., up to 10 GB RAM in our tests (Table 1). The required RAM and processing time vary based on the number of peptides evaluated, their lengths, and their three‐dimensional structures. Tables 1 and 2 document the mean RAM usage and wall clock time of the pipeline when running the basic protocol peptide datasets with 30 threads. AMPSeek requires ∼19 GB of storage as it uses container images to create the required environments for each software component (Table 3). For benchmarking, the containers were pre‐downloaded, and the download time and memory usage were not measured.

**Table 1:** Peak RAM Usage of the AMPSeek Pipeline Processes for Processing Different Datasets Using 30 Threads*^a^*

Dataset	AMPlify	LocalColabFold	tAMPer	Visualization
AMPSeek_data_10.fasta	1.3 GB	6.5 GB	1.2 GB	1.4 GB
AMPSeek_data_20.fasta	1.8 GB	6.7 GB	7 GB	1.3 GB
AMPSeek_data_100.fasta	2.4 GB	8.5 GB	8.4 GB	2.2 GB

^*a*^ The Preparation step requires negligible amounts of RAM and thus is not included in this table.

**Table 2:** Wall Clock Time of the AMPSeek Pipeline when Processing Different Datasets Using 30 Threads

Dataset	Dataset size	No. peptides	Mean peptide length	Wall clock time (hh:mm:ss)
AMPSeek_data_10.fasta	1 kB	10	121.30 aa	13:03:07
AMPSeek_data_20.fasta	3 kB	20	121.35 aa	26:14:39
AMPSeek_data_100.fasta	14 kB	100	121.33 aa	129:48:00

**Table 3:** Container Images Required to Run AMPSeek and Their Sizes*^a^*

AMPSeek step	Image	Size
RUNAMPLIFY	quay.io/biocontainers/amplify:2.0.1‐‐py36hdfd78af_0	748.68 MB
RUNCOLABFOLD	biohpc/localcolabfold:1.5	13.51 GB
RUNTAMPER	itsberkeucar/tamper:latest	4.20 GB
COMPILERESULTS	itsberkeucar/ampseek‐visualization:latest	632.04 MB

^*a*^ The image architecture for all containers is AMD64.

### Software Installation

AMPSeek is publicly distributed via GitHub (https://github.com/bcgsc/AMPSeek). The pipeline relies on Nextflow (Di Tommaso et al., 2017) as the pipeline manager and either Singularity (Kurtzer et al., 2017) or Docker (Merkel, 2014) as the container platform. We recommend that users follow the instructions listed on these software platforms’ official websites for their native operating system.

Nextflow manages the installation of the software dependencies required to run AMPSeek through Singularity. This automated approach means users are not required to perform any additional steps for software installation.

### Installing Nextflow

Nextflow installation can be done in three ways, as described in their official installation procedure (https://www.nextflow.io/docs/latest/install.html#install‐page). We recommend that users install Nextflow via the Conda package manager, which is distributed freely and can be installed using the steps explained at https://www.anaconda.com/docs/getting‐started/miniconda/install. After installing Conda, users should run the following commands to create a new environment, install Nextflow into it, and activate the environment: conda create ‐n ampseek ‐c bioconda nextflow

This environment must be activated whenever running the AMPSeek pipeline, using the command: conda activate ampseek

### Installing Singularity or Docker

Users can run the AMPSeek pipeline using either Singularity or Docker. On non‐Linux machines, we recommend that users run the pipeline with Docker, as Singularity does not natively run on them. Please refer to the installation instructions for Docker (https://docs.docker.com/engine/install/) or Singularity (https://docs.sylabs.io/guides/3.0/user‐guide/installation.html) to download a container platform depending on your system and preferences. To install Docker on MacOS machines, we recommend installing Docker Desktop (https://docs.docker.com/desktop/). To run AMPSeek on ARM64 chips with Docker, QEMU emulation (https://www.qemu.org/) is required, as the container images used only support AMD64 architecture. The following command is recommended to install QEMU emulation for Docker: docker run ‐‐privileged ‐‐rm tonistiigi/binfmt ‐‐install amd64

### Cloning AMPSeek Repository

Because AMPSeek uses Nextflow's interpreted workflow language, users simply need to clone the AMPSeek repository. Using git for this purpose is recommended. Please follow the installation instructions at https://git‐scm.com/downloads if git is not already installed on your system. git clone https://github.com/bcgsc/AMPSeek.git

The procedure will also download three shared datasets with 10, 20, and 100 peptides with a mean peptide length of 121.3 amino acids (Table 1c). These peptides are a subset derived from AMPlify's balanced and imbalanced datasets (Li et al., 2023). All the datasets can be found in the “data” folder of AMPSeek in FASTA format.

### Runtime Downloads

During runtime, AMPSeek manages the process dependencies for AMPlify, tAMPer, LocalColabfold, and the visualization step internally. For dependency management, we use containerization, which is handled in the background by Nextflow. Table 3 shows the container images that will be downloaded to the user's local storage and their specifications.

## PREDICTION OF ANTIMICROBIAL PEPTIDE ACTIVITY, THREE‐DIMENSIONAL STRUCTURE, AND TOXICITY

The basic protocol describes running AMPSeek to predict the antimicrobial bioactivity, three‐dimensional structure, and toxicity of candidate AMPs input in FASTA file format. AMPlify generates a probability‐like prediction score for each sequence, denoting its potential AMP classification. An AMPlify prediction score of 1 indicates the highest confidence of antimicrobial activity, and a score of 0 indicates the lowest confidence (Li et al., 2022). If a peptide scores above 0.5, it is predicted to be an AMP; otherwise, it is predicted not to be an AMP (Li et al., 2022). In parallel, LocalColabFold is run to predict the three‐dimensional structure of peptides. The peptide sequences and their predicted structures are then input to tAMPer to generate protein toxicity probability scores. As with the AMPlify scores, the range from 0 to 1 indicates the lowest to highest confidence that a peptide is predicted to be toxic, and a threshold of 0.5 delineates between toxic and nontoxic classifications (Ebrahimikondori et al., 2024). Finally, AMPSeek generates a report that describes and visualizes the predicted bioactivity, structure, and toxicity of the candidate AMPs.

### Necessary Resources

#### Hardware

Server or machine running a 64‐bit Linux or Mac operating system with sufficient RAM (up to 10 GB; we benchmarked our protocol on a dedicated server‐class system with 144 core Intel Xeon Gold 6254 CPU at 3.1 GHz with 2.9 TB RAM)

##### Software

AMPSeek v1.0.0 (https://github.com/bcgsc/AMPSeek)
Nextflow (https://www.nextflow.io/docs/latest/install.html#install‐page)
Docker (https://docs.docker.com/engine/install/)
Singularity (https://docs.sylabs.io/guides/3.0/user‐guide/installation.html)

##### Files

Peptide sequences in FASTA format

##### Sample data

Sample FASTA files with 10, 20, and 100 peptides are provided in the AMPSeek GitHub Repository (https://github.com/bcgsc/AMPSeek/tree/v1.0.0/data)

1 Install AMPSeek as outlined in the Strategic Planning section, and ensure that the environment is activated and/or required paths are added to the PATH environment variable. To modify your PATH variable, add the following line to the shell configuration file (e.g., .bashrc or .bash_profile), replacing /path/to/ampseek/installation below with your corresponding installation directory:

export PATH=/path/to/ampseek/installation/bin/:$PATH

2 Set up the read directory and data either manually or by download from the web:
  a. *Manually setting up the data*: Cloning AMPSeek provides three test datasets. By default, the smallest dataset, which contains 10 peptides, is the only FASTA file in the AMPSeek/data parent directory. Alternate files containing 20 and 100 peptides are located in the AMPSeek/data/alternative_data subdirectory. Data are automatically extracted from this directory when running the pipeline. For the default pipeline to work as expected, there should only be one FASTA file containing the candidate AMPs in the AMPSeek/data directory. Alternatively, if the data are stored in a different location, users can use the —data_path flag to change the location of the read directory while running AMPSeek (refer to step 3).
  b. *Downloading the data from the web with AMPSeek*: AMPSeek also allows users to input a web link to the desired peptide dataset with the —download_from flag while executing step 3. Ensure that the web link is a direct download link for a FASTA file. The system downloads the data using the specified web link and moves all of the remaining FASTA files from the AMPSeek/data directory into a backup data directory. The backup data directory will have the following naming convention: data_backup_{checksum}.
3 Run the AMPSeek Nextflow workflow with the command nextflow AMPSeek to predict the bioactivity, three‐dimensional structure, and toxicity of the target AMPs.
  For the 10‐peptide test dataset, the protocol should take ∼13 hr and require 6.5 GB of RAM (Table 1).
4 Navigate to the AMPSeek installation directory: 

cd AMPSeek

5 Set up an appropriate container:
  a. *On Linux machines*: Both Singularity and Docker are natively supported on Linux machines, allowing users to choose their preferred containerization method. We recommend using Singularity because it does not require root access for container images. Use the following command to run the pipeline with Singularity:

nextflow AMPSeek.nf ‐profile singularity

  b. *On MacOS machines*: As outlined in the Strategic Planning section, Singularity does not run natively on MacOS. For MacOS users, we recommend running AMPSeek with Docker instead. Use the following command to run AMPSeek with Docker:


nextflow AMPSeek.nf ‐profile docker

6 Check that AMPSeek has run successfully by reviewing the contents of the relevant directories.
  a. Three‐dimensional foldings: This folder contains the three‐dimensional structure prediction of peptides generated by LocalColabfold. For each peptide, there should be a ZIP file that is named after the peptide's identifier in the input FASTA. These ZIP files contain five predicted Protein Data Bank (PDB) files with three prediction cycles and Amber relaxation.
  b. tAMPer attention directories: For each peptide, the pipeline creates a folder containing self‐attention maps of amino acid residues generated during the tAMPer analysis. These maps describe how much attention the model assigns to each amino acid in the sequence.
  c. Summary HTML file: This interactive file is the final output of AMPSeek. The report provides an overview of antimicrobial and toxic peptides through summary data visualizations, followed by detailed predictions and scores for each peptide's bioactivity and toxicity in a tabular format. Each row in the table contains prediction scores alongside embedded visualizations: a scatter plot of AMPlify versus tAMPer scores (with the peptide of interest highlighted), AMPlify context attention values showing the importance of each amino acid residue in bioactivity prediction, and an interactive three‐dimensional structure plot. Users can download the complete results table as a CSV file using the button located in the top right corner.

## COMMENTARY

### Background Information

There is a pressing need to develop new antimicrobial drugs due to the growing rise of AMR. The prediction and discovery of AMPs have been streamlined in recent years, in part due to the development of *in silico* tools like AMPlify (Li et al., 2022). AMPlify can predict the bioactivity of novel peptides, which can be used to produce a list of putative AMPs to be experimentally validated. However, although many AMPs have broad‐spectrum activity, they can also demonstrate broad‐spectrum biotoxicity (Wei & Zhang, 2022). Tools like tAMPer make *in silico* toxicity prediction possible (Ebrahimikondori et al., 2024), reducing the time, cost, and labor associated with experimental screening *in vitro* and *in vivo* testing. Moreover, LocalColabFold enables three‐dimensional peptide structure prediction—these structures can be used to visualize AMPs *in silico* and are used by tAMPer for further predictions. Here, we introduce an easy‐to‐run protocol that combines the predictive power of both tools into a single command at the Unix command prompt, so they can work in tandem to generate a list of candidate AMPs that are predicted to be both bioactive and nontoxic. By packaging AMPlify, tAMPer, and LocalColabFold into a single workflow, users can run all three tools on a list of peptides with a single‐step executable. An interactive visual report is generated as the final step, providing a comprehensive summary of the characterized peptides (see Understanding Results, below). We expect this protocol to improve the ease of use of these tools and streamline *in silico* predictions for novel AMPs.

### Critical Parameters

#### Pipeline parameters

Users can provide peptide sequences downloaded from the internet with the ‐‐download_from parameter. These peptides must be in FASTA format. If the flag is not specified, the workflow is run on the FASTA file stored in the AMPSeek/data/ folder. Users can specify the output directory by using the flag ‐‐output_path and the output file name with ‐‐output_file. As different systems have different amounts of computational power available, AMPSeek allows users to specify the number of threads/CPUs (based on system configuration) with the flag ‐‐threads. The default number of threads/CPUs used for execution is 30. Even though, by default, Nextflow does not set a time cap for the process execution, for some shared computational resources, the timeout may be set to 30 min or 1 hr. We provide users the option to modify this timeout by using the ‐‐time flag. Users need to follow the format Nextflow uses for timeout specifications (https://www.nextflow.io/docs/latest/reference/process.html#process‐time). If users require a specific amount of memory allocated for their processes, they can specify the memory usage with the ‐‐mem flag. Users are expected to use Nextflow's memory specification convention (https://www.nextflow.io/docs/latest/reference/process.html#process‐memory). If ‐‐mem is not provided, the workflow will use 90% of the total available memory for each process.

The parameters for AMPlify, LocalColabFold, and tAMPer are set automatically by the AMPSeek workflow. We recommend using these parameters as they were used and optimized during the development and testing of AMPlify and tAMPer. We do not provide any command‐line flags to modify the parameters of these tools. However, users can alter the AMPSeek.nf file to change these parameters. In that case, users can refer to the following sections to understand the critical parameters of this pipeline.

#### AMPlify parameters

AMPSeek uses the default parameters of AMPlify. The only parameter that alters the predictions is ‐‐model, which controls the model weight variations to be used. AMPlify is trained on two datasets: a balanced and an imbalanced one. Based on the expected distribution of bioactivity in the input dataset, users can change this parameter. The imbalanced model is trained on data that contains a substantially higher number of inactive peptides than antimicrobial peptides, where bioactive peptides constitute ∼3.15% of the data.

#### LocalColabFold parameters

As tAMPer is dependent on LocalColabFold output, the parameters used by AMPSeek for LocalColabFold are selected to be consistent with the procedure described in tAMPer's GitHub repository. AMPSeek uses the ‐‐amber flag, which refines the predicted structures using AMBER energy minimization to find atomic coordinates that minimize the system's potential energy and maximize structural stability (Case et al., 2005). Having the ‐‐zip flag specified during the LocalColabFold run is crucial because tAMPer specifically checks for ZIP files. Otherwise, the default parameters are used for LocalColabfold. As tAMPer was trained using this configuration of LocalColabFold, it is recommended that no parameters be changed other than those specified above to generate consistent predictions.

#### tAMPer parameters

AMPSeek employs the default parameters of tAMPer. Troubleshooting parameters that change the predictions for tAMPer are dependent on the training of the model. Because AMPSeek uses the pretrained models published by Ebrahimikondori et al. (2024), changing the optimized parameters of hidden dimension size, the distance threshold for connecting two residues, or the protein embedding model type would create discrepancies and may negatively affect performance. Hence, we suggest users not change these parameters even though it is possible to run the workflow after modifying them.

AMPSeek should complete with a non‐zero exit code, and its output should show a written message that indicates a successful run. If an error occurs at any point within the workflow, it should immediately stop running. Users should carefully inspect the workflow log, output files, and error codes to identify the source(s) of error, if any. There may be problems that will result in the pipeline execution being prematurely aborted due to issues such as insufficient memory, lack of storage, or the Docker pull timeline. Refer to Table 4 for possible sources of error and their respective causes and potential solutions.

**Table 4:** Sources and Solutions to Potential Errors when Running the AMPSeek Protocol

Error	Potential source(s)	Suggested solution(s)
Pipeline execution aborted with the following error message in any of the pipeline stages: Error fetching image to cache.	Insufficient storage left in the default singularity cache directory for the images being fetched.	Change the singularity cache by exporting the environment variable NFX_SINGULARITY_CACHEDIR.
Pipeline execution aborted during visualization process with an error about the make_report.py file	There are multiple TSV files in the output directory during the execution of the visualization process. This might be due to the pipeline having terminated during an earlier run without executing the step that deletes the intermediate files generated by AMPlify—that is, the visualization process.	Remove all the TSV files in the output directory.
Pipeline cannot be executed via Docker on ARM64 chips.	QEMU emulation is required.	Install QEMU with docker run ‐‐privileged ‐‐rm tonistiigi/binfmt ‐‐install amd64
Workflow is relatively slow when running with QEMU emulation.	QEMU emulation is expected to slow the process.	We recommend using native AMD64 machines whenever possible.
Results of unintended peptides are produced without a pipeline error.	The read folder contains multiple FASTA files.	Remove the FASTA files with sequences that are not intended to be run in the workflow.
Pipeline execution aborted because a process timed out.	In high‐performance computing resources, the process timeout is set to 30 min or 1 hr.	Add ‐‐time flag to your execution by providing a new timeout value for processes.
Pipeline execution aborted due to insufficient memory.	The given memory usage is insufficient for one of the processes.	Increase the memory that each process is allowed to use with the ‐‐mem flag.
Pipeline execution aborted due to the Docker pull timeline.	The default pull timeout of 20 min is insufficient for the user's internet bandwidth.	Depending on the preferred tool (e.g., Docker), add an appropriate timeout parameter in the nextflow.config file's profiles (e.g., section.docker.pullTimeout = “amount of time”). Example: docker.pullTimeout = “1h”
Pipeline execution finished without running AMPlify, LocalColabfold, and tAMPer.	There is no FASTA file in the read folder.	Check if the read folder contains a FASTA file. If not, please move a FASTA file into the read folder.

### Understanding Results

**Figure 2:**
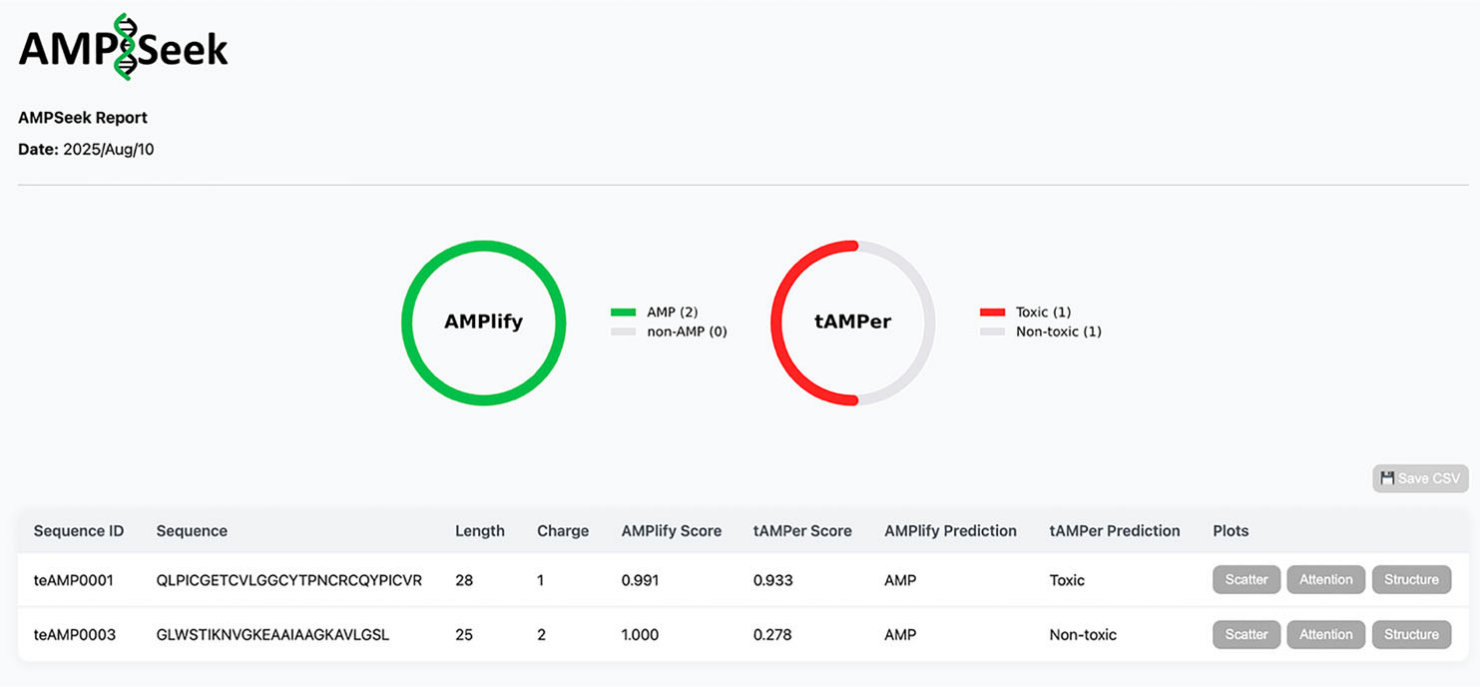
A sample AMPSeek HTML summary file output. The AMP classification and toxicity predictions of the two test sequences, teAMP001 and teAMP0003, are shown in the summary table and data visualizations

AMPSeek produces an interactive summary HTML file as its final output (Fig. 2 provides a screenshot of the report obtained when testing the AMPSeek workflow on two test sequences). The summary file displays the peptide sequences, their lengths, charges, AMPlify scores and predictions, three‐dimensional structures, and tAMPer scores and predictions. Users can optionally save these data in CSV format within the interactive HTML file.

### Author Contributions

These authors contributed equally: Emily Zhang, Berke Ucar.

**Emily Zhang**: Project administration; software; validation; manuscript writing. **Berke Ucar**: Conceptualization; data curation; methodology; project administration; software; validation; manuscript writing. **Ali Salehi**: Software; manuscript writing—review and editing. **René L. Warren**: AMPlify‐tAMPer framework idea, manuscript writing—review and editing. **Inanc Birol**: Funding acquisition; supervision; manuscript writing—review and editing.

### Conflict of Interest

The authors declare the following financial interests, which may be considered as potential competing interests: IB is a co‐founder, board member, and executive of Amphoraxe Life Sciences Inc.; AS and EZ were funded for industrial internships at Amphoraxe Life Sciences Inc. (IT44733 and IT43363, respectively). Other authors declare no competing interests that could have influenced the work reported.

## Data Availability

The data that support the protocol are openly available in the AMPSeek GitHub repository at https://github.com/bcgsc/AMPSeek/tree/v1.0.0/data.
